# Buck Converter with Cubic Static Conversion Ratio †

**DOI:** 10.3390/s24020696

**Published:** 2024-01-22

**Authors:** Delia-Anca Botila, Ioana-Monica Pop-Calimanu, Dan Lascu

**Affiliations:** Applied Electronics Department, Politehnica University Timisoara, 300223 Timișoara, Romania; ioana-m.pop@upt.ro (I.-M.P.-C.); dan.lascu@upt.ro (D.L.)

**Keywords:** buck dc–dc converter, dc analysis, static conversion ratio, component stresses, ac ripple, design equations, computer simulation

## Abstract

The paper introduces a step-down converter that exhibits a static conversion ratio of cubic nature, providing an output voltage which is much closer to the input voltage, and at the same duty cycle, compared to a wide class of one-transistor buck-type topologies. Although the proposed topology contains many components, its control is still simple, as it employs only one transistor. A dc analysis is performed, the semiconductor stresses are derived in terms of input and output voltages and output power, revealing that the semiconductor voltage stresses remain acceptable and anyway lower than in other cubic buck topology. All detailed design equations are provided. The state-space approach is used to analyze the converter in the presence of conduction losses and a procedure for calculating the individual power dissipation is provided. The feasibility of the proposed cubic buck topology is first validated by computer simulation and finally confirmed by an experimental 12 V–10 W prototype.

## 1. Introduction

In applications that require a step-down of the input voltage, buck-type topologies are the first choice due to their compact size and ability to handle varying loads [1,2]. They are commonly used in various electronic devices and applications where efficient power converters are necessary, like power supplies [3,4], battery charging [5,6,7,8,9,10,11], IoT applications [12,13], LED drivers [14,15,16] and renewable energy [17].

An interesting topology that is using the basic principle of the conventional non-isolated buck converter is presented in [18]. It contains a reduced number of components compared to the classical one due to the use of an optocoupler as a switching device and thus the diode is not used.

Another topology with a single switch, but in this case with an increased number of diodes (four), two inductors and three capacitors, and that belongs to the non-linear voltage gain family, is the single-switch semi-quadratic buck converter proposed in [19].

Also, the quadratic converter presented in [20] exhibits only one transistor, three diodes, two inductors and two capacitors. Compared to the semi-quadratic converters, the number of diodes and capacitors is decreased, and the static conversion ratio is lower than the classical buck at the same duty cycle.

In article [21], a systematic method of constructing various types of quadratic converters is introduced by inserting a three-terminal, four-element switching cell into conventional switching cells, enabling the redevelopment of known converters and proposing new topologies.

A family of single-switch PWM converters that has a wide voltage range between the input and output is presented in [22]. The proposed converters exhibit reduced current stress compared with other step-down topologies mentioned in the paper.

Some PWM switched-capacitor converters featuring a switched-capacitor-inductor cell for adjustable high step-down voltage conversion were reported in [23]. The authors performed a useful comparison with different converter topologies, while taking into account the advantages and disadvantages of each circuit.

In papers [24,25,26], different step-down topologies, where the output voltage is closer to the input voltage, are presented. Emphasizing the study of a dc conversion ratio using mathematical tools, paper [24] generalizes a two-stage stacked step-down converter to an n-stage topology. The study from [25] introduces a family of switching step-down dc–dc converters based on the principle of reduced redundant power processing. The authors in [26] present a novel switched step-down dc–dc converter suitable for applications which require a small voltage difference between input and output. The converter demonstrates a measured efficiency of approximately 90% over a wide duty cycle range.

The authors from [27] present a single-input, double-output, unidirectional and bidirectional dc–dc buck converter. The paper shows the control strategy, PWM implementation and design specifications. A dual-output converter integrating two inductors into one magnetic coil, which is claimed to have a good dynamic behavior, is proposed in [28]. Another paper, [29], introduces a dual-output, high step-down converter with six active switches that has the advantages of improved efficiency through zero-voltage switching, compatibility with an existing buck controller integrated circuit, the ability to control multiple output voltages with a single controller and can be extended to more than two output rails.

A multiphase, synchronous buck converter is introduced in [30]. This converter employs two types of fully integrated current sensing schemes, as described in the paper.

The interleaved converter with coupled inductors from [31] has three windings built within a single core. The advantages of this converter, over other topologies, are also presented in the paper. A high step-down dc–dc converter with continuous output current, which maintains the soft switching of the semiconductors through a broad power range and reduces the MOSFET peak current with coupled inductors and series capacitors, is introduced in [32].

The authors of [33] analyzed some multilevel cascaded dc–dc converters for photovoltaic applications, together with some half-bridge topologies. One of those circuits is the multilevel cascaded dc–dc buck converter and another one is represented by the half-bridge buck converter. The main conclusion resulting from the paper is that, for series operation of photovoltaic generators, the buck topology is a proper choice, and, in the cascaded photovoltaic systems, the half-bridge topology of the buck converter is preferred. Another cascaded buck converter that improved efficiency by repositioning the second-stage inductor, reducing the total volume and conduction losses of the magnetic components, is introduced in [34].

An extended and comprehensive comparison of a variety existing step-down topologies, from which it can be remarked that cubic buck converters are not very common, is presented in [35]. In [36], the authors introduced a single-switch cubic buck converter and they recommend to use this topology for low power loads.

The present paper introduces a new cubic buck-type topology. It extends the authors’ previous research [37], where an ideal buck-boost topology of cubic nature is theoretically analyzed and verified only at a computer simulation level. Starting from the same parent converter [38], similar to the one from [37], the authors propose a cubic topology, this time of step-down nature, which is obtained using the same switching cell method. The content of the present paper is related to aspects that refer to:

- The dc analysis for the ideal converter, together with the semiconductor stresses and ripples calculation;
- The expression of the duty cycle for which a conversion from a given input voltage V_g_ to a desired output voltage V_o_ is achieved;
- A comprehensive comparison of the proposed converter with other buck-type topologies, revealing superior features, at least compared to the cubic topologies;
- The continuous conduction mode (CCM) operation conditions for a proper design of the inductors;
- The analysis of the non-ideal converter in the presence of conduction losses with the help of the state-space matrices. The abovementioned, together with the design equations, can be found in Section 2;
- A simulation for validating the theoretical operation of the ideal converter, as well as the experimental results on a 12 V–10 W prototype all confirm the feasibility of the proposed topology. These aspects are presented in Section 3;
- The discussions and conclusions, which are included in Section 4 and Section 5, respectively.

## 2. Materials and Methods

The proposed cubic buck converter was derived from the cubic boost topology presented in [38]. Starting from its schematic, the proposed circuit was obtained by applying the three terminals switching cell rotation technique [39].

**Figure 1:**
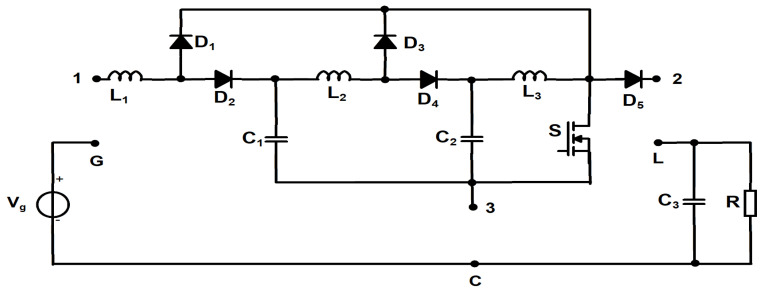
The cubic boost converter from [38]—switching cell extraction.

Basically, this method implies that the cell containing the switching devices and the reactive elements has three terminals which are connected to the supply voltage terminal (G), load terminal (L) and to the common point (C) of the circuit, as displayed in Figure 1. After the cell is extracted from the original converter, the semiconductors are swapped with single-pole single-throw (SPST) switches, maintaining the same control sequence. The next step is to obtain new circuits by rotating the resulting cell between the terminals G, L, C. Finally, after performing all five possible cell rotations, for each resulting topology, switch synthesis is performed, and the SPST switches are replaced by the corresponding semiconductor devices.

**Figure 2:**
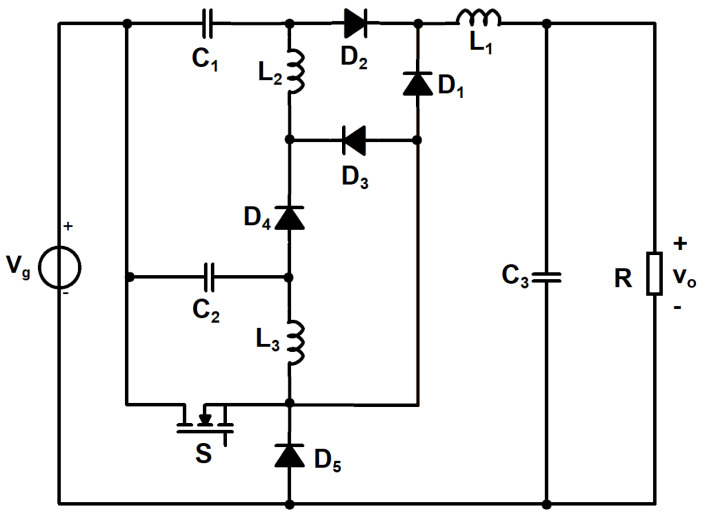
The proposed cubic buck topology.

As a consequence, by applying the technique, which was previously described, the proposed converter, in its final form, is obtained for the combination 1-L, 2-C, 3-G connection and its schematic is presented in Figure 2. This new converter is first analyzed assuming ideal components, invoking the small ripple assumption for inductor currents and capacitor voltages and CCM operation. The transistor switching function q(t) is a pulse width modulated (PWM) signal of duty cycle D and switching frequency f_S_ corresponds to the switching period T_S_.

**Figure 3:**
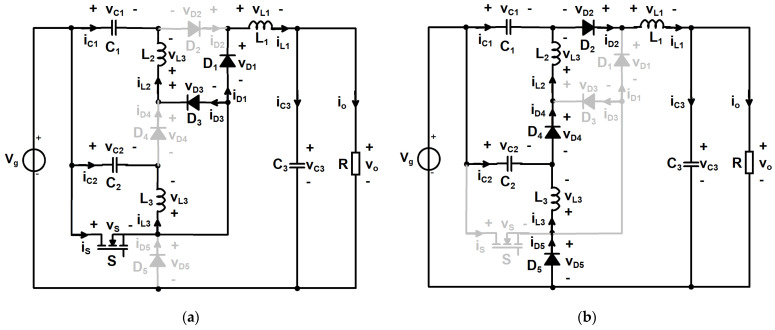
Topological states of the proposed converter, revealing the conducting devices: (**a**) first topological state; (**b**) second topological state.

In the first topological state, which extends from 0 to D∙T_S_, the transistor conducts alongside diodes D_1_ and D_3_. The remaining amount of time until reaching a complete switching period, which is from D∙T_S_ to T_S_, corresponds to the second topological state, in which the transistor is off and only diodes D_2_, D_4_ and D_5_ are forward biased and conducting. The schematics associated to each topological state are depicted in Figure 3.

In order to apply the volt-second balance principle to obtain the dc capacitor voltages, the dc voltage across each inductor shall be determined from both topological states. It results that:

(1) $$D\cdot \left(V_{g}-V_{C3}\right)+\left(1-D\right)\cdot \left(V_{g}-V_{C3}-V_{C1}\right)=0$$

(2) $$D\cdot V_{C1}+\left(1-D\right)\cdot \left(V_{C1}-V_{C2}\right)=0$$

(3) $$D\cdot V_{C2}+\left(1-D\right)\cdot \left(-V_{g}+V_{C2}\right)=0$$

The capacitor currents are determined in each topological state and thus, the charge balance principle can be used to derive the dc inductor currents values:

(4) $$D\cdot (-I_{L2})+\left(1-D\right)\cdot (I_{L1}-I_{L2})=0$$

(5) $$D\cdot (-I_{L3})+\left(1-D\right)\cdot (I_{L2}-I_{L3})=0\;$$

(6) $$D\cdot \left(I_{L1}-\frac{V_{C3}}{R}\right)+\left(1-D\right)\cdot \left(I_{L1}-\frac{V_{C3}}{R}\right)=0\;$$

Therefore, from (1) to (3), the dc capacitor voltages are obtained, along with the static conversion ratio:

(7) $$V_{C1}={\left(1-D\right)}^{2}\cdot V_{g}\;$$

(8) $$V_{C2}=\left(1-D\right)\cdot V_{g}$$

(9) $$V_{C3}=V_{o}=D\cdot (D^{2}-3\cdot D+3)\cdot V_{g}$$

(10) $$M=\frac{V_{o}}{V_{g}}=D\cdot (D^{2}-3\cdot D+3)=1-{\left(1-D\right)}^{3}$$

For a given conversion from an input voltage V_g_ to a desired output voltage V_o_, according to (10), the necessary duty cycle is:

(11) $$D=1-\sqrt[{3}]{1-\frac{V_{o}}{V_{g}}}$$

**Figure 4:**
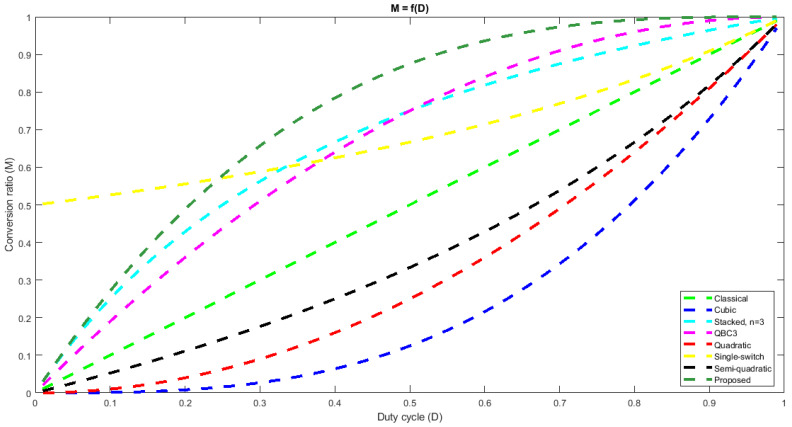
Static conversion ratio vs. duty cycle comparison between different buck-type converters and the proposed cubic buck topology: Classical [1](light green); Cubic [36] (dark blue); Stacked, n = 3 [24] (light blue); QBC3 [25] (magenta); Quadratic [20] (red); Single-switch [26] (yellow); Semi-quadratic [19] (black); Proposed (dark green).

Equation (10) describes a static conversion ratio that is typical to a step-down converter topology, as it is always less than unity. In order to better show the step-down nature of the proposed circuit, its static conversion ratio dependency on the duty cycle is depicted in Figure 4, together with the ones corresponding to the classical buck [1], cubic buck [36], stacked buck [24], QBC3 [25], the quadratic buck [20], single-switch buck [26] and the semi-quadratic buck [19] converters.

Starting from this representation, it can be observed that, for any duty cycle, the proposed topology exhibits the highest static conversion ratio compared to the all of the mentioned converters. This feature ensures that the proposed cubic buck converter can be used in various applications where a low step-down of the input voltage is needed.

From Equations (4) to (6), the dc inductor currents are obtained, using also the dc capacitor voltages previously derived:

(12) $$I_{L1}=D\cdot \left(D^{2}-3\cdot D+3\right)\cdot \frac{V_{g}}{R}\;$$

(13) $$I_{L2}=D\cdot \left(1-D\right)\cdot \left(D^{2}-3\cdot D+3\right)\cdot \frac{V_{g}}{R}$$

(14) $$I_{L3}=D\cdot {\left(1-D\right)}^{2}\cdot \left(D^{2}-3\cdot D+3\right)\cdot \frac{V_{g}}{R}$$

It has to be mentioned that using (11) and replacing R by $\frac{V_{o}^{2}}{P_{o}}$ , the dc capacitor voltages and inductor currents can be expressed only in terms of V_g_, V_o_ and P_o_. This is valid also for the dc semiconductor currents and voltage stresses that will be further derived.

**Figure 5:**
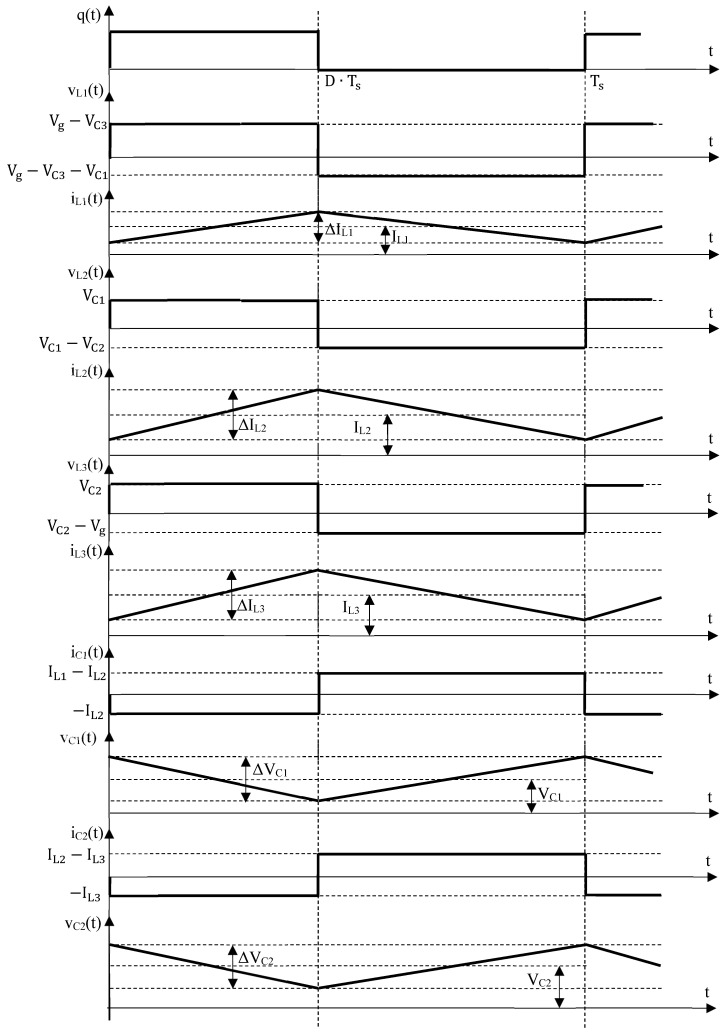
Theoretical waveforms for the reactive elements of the proposed cubic buck converter.

The theoretical waveforms corresponding to the reactive elements are depicted in Figure 5.

In a dc–dc converter design process, in order to be able to choose the suitable devices, a key-role is played by the semiconductor stresses. The voltage stress is given by the topological state in which the respective device is in the off-state, whereas the dc current stress is obtained from its on-state. The final values of the semiconductor stresses result from (7) to (9) and (12) to (14) as:

(15) $$V_{S}=V_{g}$$

(16) $$I_{S}=D\cdot (I_{L1}+I_{L2}+I_{L3})=D^{2}\cdot {\left(D^{2}-3\cdot D+3\right)}^{2}\cdot \frac{V_{g}}{R}=\frac{P_{o}}{V_{g}}$$

(17) $$V_{D1}=-V_{C1}+V_{g}=D\cdot \left(2-D\right)\cdot V_{g}=V_{g}\cdot \left(1-\sqrt[{3}]{{\left(1-M\right)}^{2}}\right)$$

(18) $$I_{D1}=D\cdot I_{L1}=D^{2}\cdot \left(D^{2}-3\cdot D+3\right)\cdot \frac{V_{g}}{R}=\frac{P_{o}}{V_{o}}\cdot \left(1-\sqrt[{3}]{1-M}\right)\;$$

(19) $$V_{D2}=V_{C1}={\left(1-D\right)}^{2}\cdot V_{g}=V_{g}\cdot \sqrt[{3}]{{\left(1-M\right)}^{2}}$$

(20) $$I_{D2}=\left(1-D\right)\cdot I_{L1}=D\cdot \left(1-D\right)\cdot \left(D^{2}-3\cdot D+3\right)\cdot \frac{V_{g}}{R}=\frac{P_{o}}{V_{o}}\cdot \sqrt[{3}]{1-M}$$

(21) $$V_{D3}=-V_{C2}+V_{g}=D\cdot V_{g}=V_{g}\cdot \left(1-\sqrt[{3}]{1-M}\right)\;$$

(22) $$I_{D3}=D\cdot I_{L2}=D^{2}\cdot \left(1-D\right)\cdot \left(D^{2}-3\cdot D+3\right)\cdot \frac{V_{g}}{R}=\frac{P_{o}}{V_{o}}\cdot \left(1-\sqrt[{3}]{1-M}\right)\cdot \sqrt[{3}]{1-M}$$

(23) $$V_{D4}=V_{C2}=\left(1-D\right)\cdot V_{g}=V_{g}\cdot \sqrt[{3}]{1-M}$$

(24) $$I_{D4}=\left(1-D\right)\cdot I_{L2}=D\cdot {\left(1-D\right)}^{2}\cdot \left(D^{2}-3\cdot D+3\right)\cdot \frac{V_{g}}{R}=\frac{P_{o}}{V_{o}}\cdot \sqrt[{3}]{{\left(1-M\right)}^{2}}$$

(25) $$V_{D5}=V_{g}$$

(26) $$I_{D5}=\left(1-D\right)\cdot I_{L3}=D\cdot {\left(1-D\right)}^{3}\cdot \left(D^{2}-3\cdot D+3\right)\cdot \frac{V_{g}}{R}=\frac{P_{o}}{V_{o}}\cdot \left(1-M\right)\;$$

**Figure 6:**
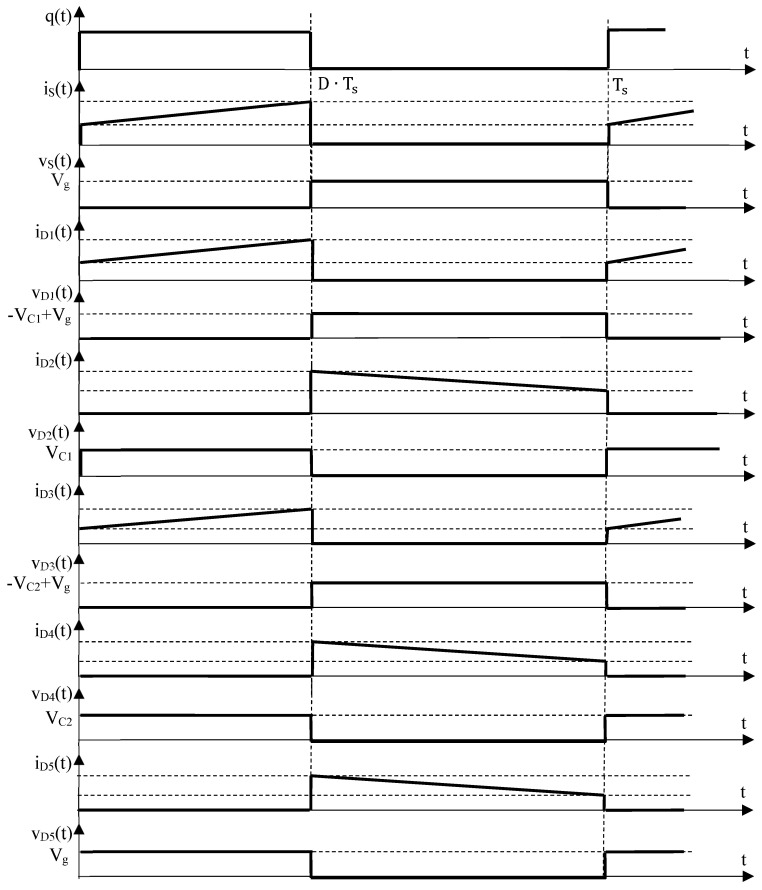
Theoretical waveforms for the semiconductor devices of the proposed cubic buck converter.

The waveforms associated to the semiconductor devices are sketched in Figure 6, where the voltage stresses are also revealed neglecting the capacitor voltage ripples.

The proposed topology is compared to several other relevant buck-type converters reported in the literature. Table 1 summarizes this comparison revealing the main merit parameters. For a fair comparison, the investigated topologies are all one transistor converters. As expected, as converter order is increasing, the total number of components is increasing too. It can be observed that the proposed topology has the same number of components like the cubic converter in [36] and only one more component compared to the stacked converter reported in [24]. Regarding the number of diodes, the proposed converter is the one with the highest number, together with the cubic [36]. However, in spite of being a cubic topology, the system order is not the highest one, as the stacked [24] is of eighth order. If the static conversion ratio is under discussion, Figure 4 reveals that the proposed converter provides the highest static conversion ratio of all converters at the same duty ratio in the usual duty cycle range.

Transistor dc current stress is very good, as it is lower than the classical [1], cubic [36], quadratic [20] and semi-quadratic [19] and the same as the QBC3 [25]. A comparison to the stacked [24] is irrelevant, as n denotes the number of stages which is an additional parameter that is not present in the other topologies. It can be then concluded that the proposed cubic converter assures the lowest current stress of the transistor. Transistor voltage stress is the same to the classical [1], QBC3 [25], but lower than cubic [36], and quadratic [20] and only the stacked [24], single switch [26] and semi-quadratic [19] operate with a better transistor voltage stress. Hence it can be stated that transistor voltage stress is moderate and better than the cubic [36] counterpart. The same considerations hold for the diode dc current stress that is superior to the cubic [36], stacked [24], QBC3 [25], quadratic [20] and semi-quadratic [19]. Regarding the maximum diode voltage stress, it is equal to the classical [1], QBC3 [25], quadratic [20], higher than semi-quadratic [19], single switch [26] and lower than the cubic [36]. It can be observed that all the merit parameters of the proposed converter are better than the cubic [36], which is an important feature.

**Figure 7:**
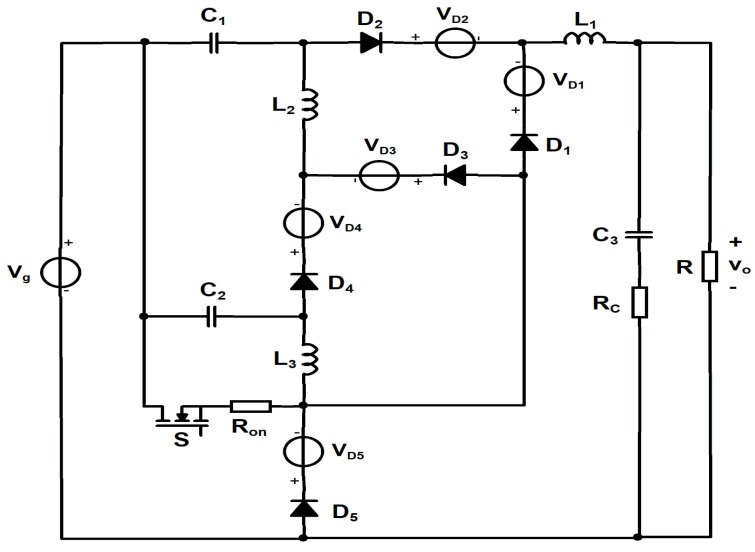
Proposed cubic buck converter including the lossy elements.

As it is known, the conduction losses could significantly modify and influence the static characteristics, including converter efficiency. Therefore, a dc analysis in the presence of losses is compulsory. As the classical dc analysis techniques are difficult to apply in the presence of the losses, the state-space approach is used. The schematic including the conduction losses is presented in Figure 7. The loss elements are the transistor on resistance, R_on_, the output capacitor equivalent series resistance R_C_ and the forward voltage diodes drops V_D1_, V_D2_, V_D3_, V_D4_ and V_D5_.

The state vector, x, includes the capacitor voltages and the inductor currents. The output vector, y, contains the output voltage, while the input vector, u, consists of the input voltage and the forward voltage drops of the diodes. With these definitions, the resulting state matrices are:

(27) $$\left\{\begin{array}{c} A_{1}=\left[\begin{array}{cccccc} -\frac{R_{\mathrm{on}}}{L_{1}} & -\frac{R_{\mathrm{on}}}{L_{1}} & -\frac{R_{\mathrm{on}}}{L_{1}} & 0 & 0 & -\frac{R}{\left(R+R_{C}\right)\cdot L_{1}}\\ -\frac{R_{\mathrm{on}}}{L_{2}} & -\frac{R_{\mathrm{on}}}{L_{2}} & -\frac{R_{\mathrm{on}}}{L_{2}} & \frac{1}{\;L_{2}} & 0 & 0\\ -\frac{R_{\mathrm{on}}}{L_{3}} & -\frac{R_{\mathrm{on}}}{L_{3}} & -\frac{R_{\mathrm{on}}}{L_{3}} & 0 & \frac{1}{\;L_{3}} & 0\\ 0 & -\frac{1}{C_{1}} & 0 & 0 & 0 & 0\\ 0 & 0 & -\frac{1}{C_{2}} & 0 & 0 & 0\\ \frac{1}{C_{3}} & 0 & 0 & 0 & 0 & -\frac{1}{\left(R+R_{C}\right)\cdot C_{3}} \end{array}\right],\;\;\;B_{1}=\left[\begin{array}{cccccc} \frac{1}{\;L_{1}} & -\frac{1}{\;L_{1}} & 0 & 0 & 0 & 0\\ 0 & 0 & 0 & -\frac{1}{\;L_{2}} & 0 & 0\\ 0 & 0 & 0 & 0 & 0 & 0\\ 0 & 0 & 0 & 0 & 0 & 0\\ 0 & 0 & 0 & 0 & 0 & 0\\ 0 & 0 & 0 & 0 & 0 & 0 \end{array}\right],\\ E_{1}=\left[\begin{array}{cccccc} 0 & 0 & 0 & 0 & 0 & \frac{R}{R+R_{C}} \end{array}\right],\;\;\;\;\;\;\;\;F_{1}=\left[\begin{array}{c} 0\\ 0\\ 0\\ 0\\ 0\\ 0 \end{array}\right] \end{array}\right.$$

(28) $$\left\{\begin{array}{c} A_{2}=\left[\begin{array}{cccccc} 0 & 0 & 0 & -\frac{1}{\;L_{1}} & 0 & -\frac{R}{\left(R+R_{C}\right)\cdot L_{1}}\\ 0 & 0 & 0 & \frac{1}{\;L_{2}} & -\frac{1}{\;L_{2}} & 0\\ 0 & 0 & 0 & 0 & \frac{1}{\;L_{3}} & 0\\ \frac{1}{C_{1}} & -\frac{1}{C_{1}} & 0 & 0 & 0 & 0\\ 0 & \frac{1}{C_{2}} & -\frac{1}{C_{2}} & 0 & 0 & 0\\ \frac{1}{C_{3}} & 0 & 0 & 0 & 0 & -\frac{1}{\left(R+R_{C}\right)\cdot C_{3}} \end{array}\right],\;\;\;B_{2}=\left[\begin{array}{cccccc} \frac{1}{\;L_{1}} & 0 & -\frac{1}{\;L_{1}} & 0 & 0 & 0\\ 0 & 0 & 0 & 0 & -\frac{1}{\;L_{2}} & 0\\ -\frac{1}{\;L_{3}} & 0 & 0 & 0 & 0 & -\frac{1}{\;L_{3}}\\ 0 & 0 & 0 & 0 & 0 & 0\\ 0 & 0 & 0 & 0 & 0 & 0\\ 0 & 0 & 0 & 0 & 0 & 0 \end{array}\right],\\ E_{2}=\left[\begin{array}{cccccc} 0 & 0 & 0 & 0 & 0 & \frac{R}{R+R_{C}} \end{array}\right],\;\;\;\;\;\;\;\;F_{2}=\left[\begin{array}{c} 0\\ 0\\ 0\\ 0\\ 0\\ 0 \end{array}\right] \end{array}\right.$$

The static conversion ratio of the proposed cubic buck-boost converter is obtained as:

(29) $$M=\mathrm{FD}-\mathrm{ED}\cdot {\mathrm{AD}}^{-1}\cdot \mathrm{BD}$$

where:

(30) $$\mathrm{AD}=D\cdot A_{1}+\left(1-D\right)\cdot A_{2}$$

(31) $$\mathrm{BD}=D\cdot B_{1}+\left(1-D\right)\cdot B_{2}$$

(32) $$\mathrm{ED}=D\cdot E_{1}+\left(1-D\right)\cdot E_{2}$$

(33) $$\mathrm{FD}=D\cdot F_{1}+\left(1-D\right)\cdot F_{2}$$

If Equation (29) is evaluated with zero lossy elements, the value of the static conversion ratio is the same as in (10), confirming the validity of the ideal static conversion ratio value. Also, the converter efficiency can be calculated using the state-space approach simply by including the input current in the output vector. Moreover, individual component losses can be found extending the output vector with the diode currents and using the dc inductor currents calculated in the state vector.

To ensure a proper converter design, the ripples of the inductor currents and capacitor voltages are necessary. Equations (7)–(9) and (12)–(14) are used for their calculation in the ideal case and the final results are:

(34) $${\Delta I}_{L1}=\frac{D\cdot \left(V_{g}-V_{C3}\right)}{L_{1}\cdot f_{S}}=\frac{D\cdot {\left(1-D\right)}^{3}\cdot V_{g}}{L_{1}\cdot f_{S}}$$

(35) $${\Delta I}_{L2}=\frac{D\cdot V_{C1}}{L_{2}\cdot f_{S}}=\frac{D\cdot {\left(1-D\right)}^{2}\cdot V_{g}}{L_{2}\cdot f_{S}}$$

(36) $${\Delta I}_{L3}=\frac{D\cdot V_{C2}}{L_{3}\cdot f_{S}}=\frac{D\cdot \left(1-D\right)\cdot V_{g}}{L_{3}\cdot f_{S}}$$

(37) $${\Delta V}_{C1}=\frac{\left(1-D\right)\cdot \left(I_{L1}-I_{L2}\right)}{C_{1}\cdot f_{S}}=D^{2}\cdot \left(1-D\right)\cdot \left(D^{2}-3\cdot D+3\right)\cdot \frac{V_{g}}{C_{1}\cdot R\cdot f_{S}}$$

(38) $${\Delta V}_{C2}=\frac{\left(1-D\right)\cdot \left(I_{L2}-I_{L3}\right)}{C_{2}\cdot f_{S}}=D^{2}\cdot {\left(1-D\right)}^{2}\cdot \left(D^{2}-3\cdot D+3\right)\cdot \frac{V_{g}}{C_{2}\cdot R\cdot f_{S}}$$

(39) $${\Delta V}_{C3}=\frac{{\Delta I}_{L1}}{8\cdot C_{3}\cdot f_{S}}=D\cdot \left(D^{2}-3\cdot D+3\right)\cdot \frac{V_{g}}{32\cdot C_{3}\cdot R\cdot f_{S}}$$

In order to be sure that the converter operates in CCM, the minimum value of the current through each diode needs to be positive when it conducts. Hence, the imposed conditions for the CCM operation result as follows:

(40) $${\mathrm{diodes}\;D}_{1},D_{2}:\frac{2\cdot L_{1}\cdot f_{S}}{R}\geq \frac{{\left(1-D\right)}^{3}}{D^{2}-3\cdot D+3}$$

(41) $${\mathrm{diodes}\;D}_{3},D_{4}:\frac{2\cdot L_{2}\cdot f_{S}}{R}\geq \frac{1-D}{D^{2}-3\cdot D+3}$$

(42) $${\mathrm{diode}\;D}_{5}:\frac{2\cdot L_{3}\cdot f_{S}}{R}\geq \frac{1}{\left(1-D\right)\cdot \left(D^{2}-3\cdot D+3\right)}$$

The reactive elements design is based on fulfilling the small ripple conditions. In case of inductor currents, the ripple is limited to a maximum of 25% of the respective dc current value. For the internal capacitor voltages, the ripples are restricted to being lower than 10% of the dc voltage, while the ripple of the output capacitor voltage is set to a maximum limit of 5% of its corresponding dc value. Taking into account these considerations, from (7) to (9), (12) to (14) and (34) to (39), the minimum values of the reactive components are:

(43) $$L_{1}\geq \frac{4\cdot R\cdot {\left(1-D\right)}^{3}}{\left(D^{2}-3\cdot D+3\right)\cdot f_{S}}$$

(44) $$L_{2}\geq \frac{4\cdot R\cdot \left(1-D\right)}{\left(D^{2}-3\cdot D+3\right)\cdot f_{S}}$$

(45) $$L_{3}\geq \frac{4\cdot R}{\left(1-D\right)\cdot \left(D^{2}-3\cdot D+3\right)\cdot f_{S}}$$

(46) $$C_{1}\geq \frac{10\cdot D^{2}\cdot \left(D^{2}-3\cdot D+3\right)}{R\cdot f_{S}\cdot \left(1-D\right)}$$

(47) $$C_{2}\geq \frac{10\cdot D^{2}\cdot \left(1-D\right)\cdot \left(D^{2}-3\cdot D+3\right)}{R\cdot f_{S}}\;$$

(48) $$C_{3}\geq \frac{5}{8\cdot R\cdot f_{S}}$$

In the above equations, the duty cycle is known from the static conversion ratio.

Design example of the proposed cubic buck converter:

- Input voltage: V_g_ = 15 V;
- Output voltage: V_o_ = 12 V;
- Output power: P_o_ = 10 W;
- Switching frequency: f_s_ = 100 kHz.

These design parameters are allowing the immediate calculation of the output resistor and static conversion ratio values, resulting in R = 14.4 Ω and M = 0.8. Afterwards, from (10), a duty cycle of D = 0.4151 is obtained.

The minimum inductor values that assure CCM operation result from (40) to (42) as L_1min_ = 3.26 μH, L_2min_ = 9.53 μH and L_3min_ = 27.86 μH. However, the adopted values of the reactive elements are based on the small ripple assumption, which in case of inductors, is a stronger condition. They are those in (43)–(48), and the final values that are used in the simulation and experimental parts were selected as follows: L_1_ = 100 μH, L_2_ = 220 μH, L_3_ = 820 μH, C_1_ = 10 μF, C_2_ = 2.2 μF and C_3_ = 3.3 μF. Also, the semiconductor devices are chosen after evaluating the stresses described in Equations (15)–(26), which result in I_S_ = 0.6665 A, I_D1_ = 0.3459 A, I_D2_ = 0.4874 A, I_D3_ = 0.2023 A, I_D4_ = 0.2851 A, I_D5_ = 0.1667 A, V_S_ = V_D5_ = 15 V, V_D1_ = 9.8684 V, V_D2_ = 5.1316 V, V_D3_ = 6.2265 V and V_D4_ = 8.7735 V.

According to these values, the selected devices are specified in Section 3.2.

## 3. Results

### 3.1. Simulation Results

**Figure 8:**
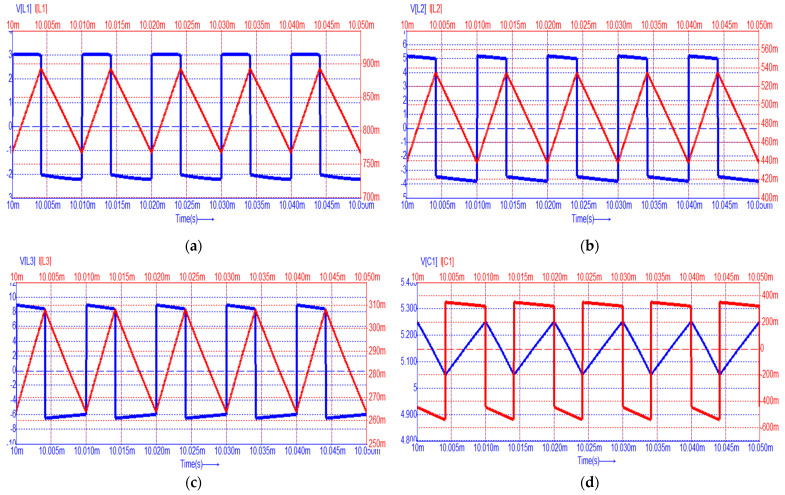

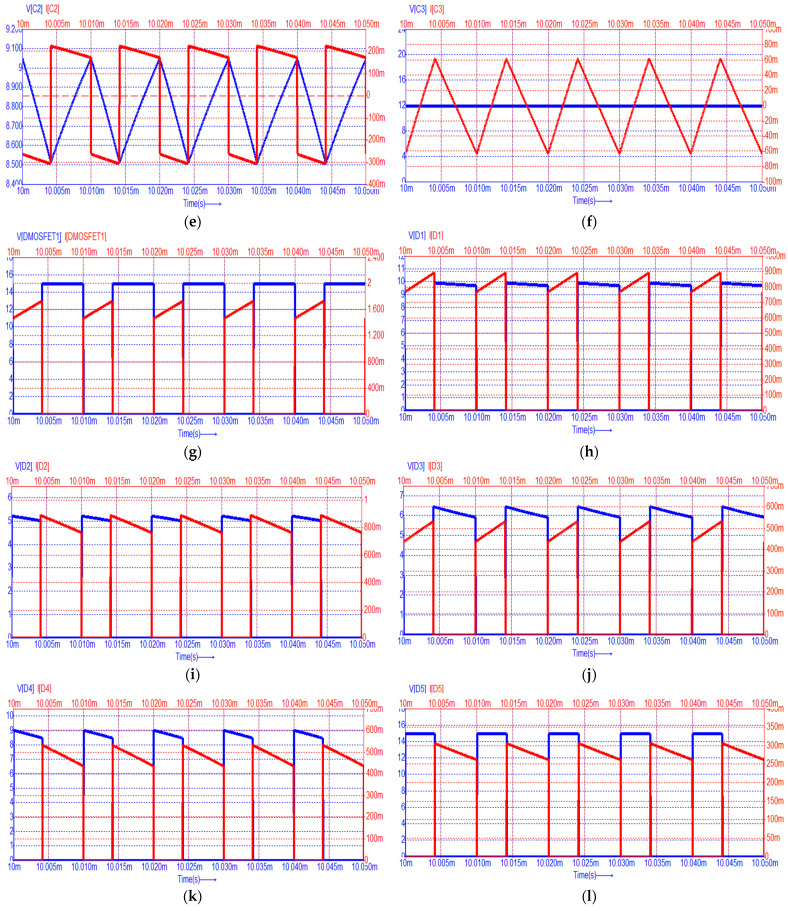
Simulation results (voltages with blue and currents with red): (**a**) for inductor L_1_; (**b**) for inductor L_2_; (**c**) for inductor L_3_; (**d**) for capacitor C_1_; (**e**) for capacitor C_2_; (**f**) for capacitor C_3_; (**g**) for transistor S; (**h**) for diode D_1_; (**i**) for diode D_2_; (**j**) for diode D_3_; (**k**) for diode D_4_; (**l**) for diode D_5_.

For a first validation of the theoretical considerations, the operation of the proposed cubic buck converter was simulated using the Caspoc [40] simulation tool. All components are assumed to be ideal, with the values that were obtained from the design example. The results are shown in Figure 8, where the current through each device is drawn in red and the voltage across it in blue.

Inspecting the simulation results showed above, it can be observed that the inductor voltages exhibit a rectangular shape, while their currents are triangular, as expected. Quantitatively speaking, the dc values, the peak-to-peak ripples for the inductor currents and the capacitor voltages match to those theoretically predicted, as Figure 5 and Figure 6 reveal compared to Equations (7)–(9) and (12)–(26). The same agreement stands for the dc semiconductor currents and their voltage stresses. Overall, the ideal operation of the proposed converter is validated, and this allows for the next step of experimental validation.

### 3.2. Experimental Results

For the experimental validation, a prototype of the proposed cubic buck converter was built using the IRLB8721 transistor and the RFN10NS6S diode for all diodes in the circuit. Afterwards, because of its floating connection, the transistor was controlled by a circuit that involved the usage of an optocoupler device.

**Figure 9:**
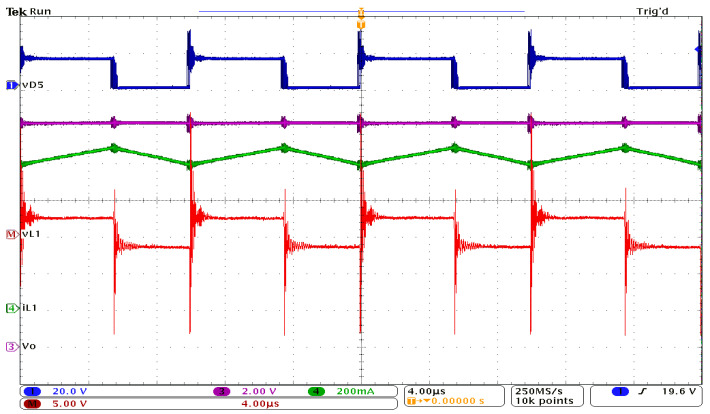
Experimental waveforms: reference signal, vD5 (dark blue); inductor L_1_ voltage, vL1 (red); current through L_1_, iL1 (green); output voltage Vo (purple).

**Figure 10:**
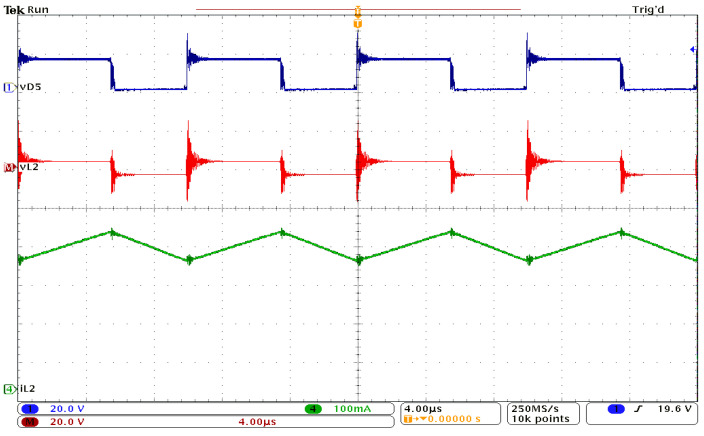
Experimental waveforms: reference signal, vD5 (dark blue); inductor L_2_ voltage, vL2 (red); current through L_2_, iL2 (green).

**Figure 11:**
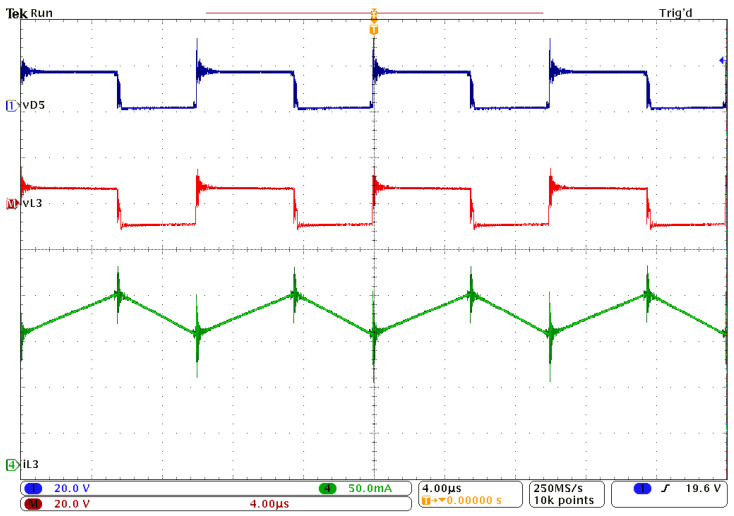
Experimental waveforms: reference signal, vD5 (dark blue); inductor L_3_ voltage, vL3 (red); current through L_3_, iL3 (green).

Figure 9 presents the waveforms obtained for the inductor L_1_ and the output voltage. Figure 10 shows the experimental results for inductor L_2_, while Figure 11 depicts the current and voltage waveforms for inductor L_3_. In all cases, the voltage across diode D_5_ is used as a reference signal. All these waveforms qualitatively confirm the theoretical and simulation results. The spikes accompanying the voltage measurements are due to the fact that a breadboard was used, and the printed circuit board (PCB) was not optimized, as the purpose was to experimentally sustain the feasibility of the proposed topology.

Two cases have been examined with the help of an electronic load operated either as a constant resistor or as a variable resistor.

- Case 1: different values of the duty cycle and constant output resistor value;
- Case 2: different resistor values while keeping a constant output voltage.

**Figure 12:**
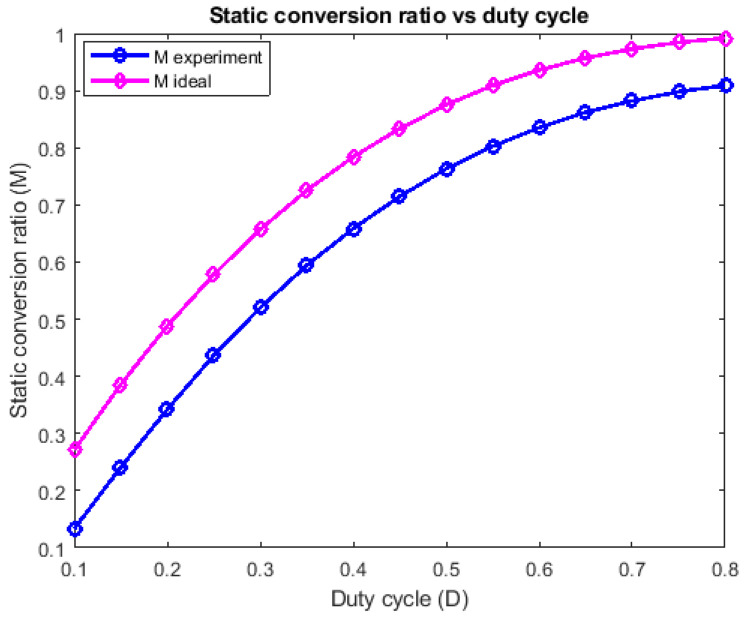
Comparison of the ideal and experimental static conversion ratio against the duty cycle, with constant load resistance.

**Figure 13:**
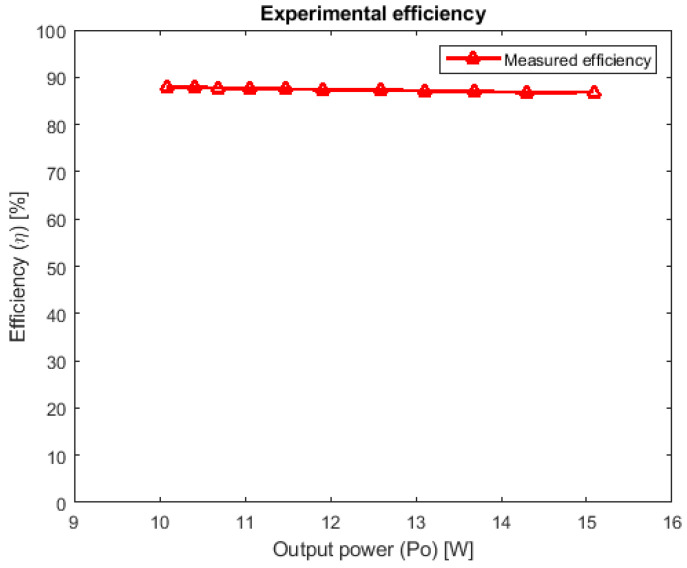
Experimental efficiency against output power.

In case 1, the measurements were conducted in order to derive a graphical representation for comparing the ideal and experimental static conversion ratios, results which are shown in Figure 12. In case 2, the experimental efficiency dependency on the output power was studied and it can be seen in Figure 13.

With respect to Figure 12, the measured characteristic exhibits the same shape as the ideal one, but it is shifted downwards. This shift is due to the non-zero voltage drop of the five diodes, their influence being more significant than that of the transistor on resistance. As Figure 13 reveals, the efficiency achieves high values at moderate output power and its flat allure is due to the fact that the number of conducting devices in each topological state is the same (one transistor and two diodes in the first one and three diodes in the second one). As the duty cycle increases, the conduction time of the transistor increases too and also all inductor currents. As a consequence, the power dissipation across the transistor increases, becoming the main power loss, while the total power dissipated by the diodes remains somehow constant, because two diodes are conducting in the first topological state and three in the second one, which is becoming shorter at higher duty cycles. This qualitatively explains why the efficiency is slightly getting lower for high duty cycles. Regardless, the efficiency exceeds 85% on the investigated power range with a small variation with respect to the output power, as seen in Figure 13.

## 4. Discussion

The paper proposes a single-transistor cubic buck topology. After comparing its operation to a set of seven buck-type converters, it is concluded that, at the same duty cycle, the proposed converter exhibits the highest static conversion ratio; hence, it provides the highest output voltage for the same supply and control. This means that the proposed topology is able to provide a small step-down at moderate duty cycles. Therefore, its practical use will refer to applications in which an output voltage lower, but still close to the input voltage is needed. Such possible applications could be in automotive industry or in renewable energy appliances.

Regarding the transistor current stress, it is the lowest from the set of converters that were analyzed, and all the merit parameters are superior to the known cubic topology. As expected, in the real lossy converter the static conversion ratio is lower compared to the ideal case, but in spite of the fact that five diodes are present, the efficiency is maintained higher than 85% in a reasonable power range.

The CCM operation conditions are derived, together with the peak-to-peak ripples for the inductor currents and capacitor voltages. Using them, a set of equations for designing the inductors and the capacitors are provided. Also, the semiconductor components are chosen based on the device current and voltage stresses.

Simulation and practical experiments confirm all the theoretical considerations with respect to dc analysis, ac analysis, waveforms and design equations.

## 5. Conclusions

In general, higher order converters proposed in the literature have been limited to quadratic converters, while cubic static conversion ratios are rarely encountered. This paper tries to overpass this barrier and proposes a cubic buck topology that exhibits a higher static conversion ratio than that of the classical buck [1], cubic buck [36], stacked buck [24], QBC3 [25], the quadratic buck [20], single-switch buck [26] and the semi-quadratic buck [19] converters, at the same duty cycle. Compared to the other previously reported cubic topology, the new converter has the same complexity, while exhibiting lower transistor and diodes stresses. In spite of its cubic nature, for a certain application, the duty cycle can be easily determined together with component stresses and high efficiencies are obtained in the investigated power range.

Future research will focus on deriving and analyzing other cubic topologies and exploit their facilities in different applications.

## Figures and Tables

**Table 1:** Comparison between the proposed converter and some other step-down topologies.

Parameter	Classical [1]	Cubic [36]	Stacked [24]	QBC3 [25]	Quadratic [20]	Single Switch [26]	Semi-Quadratic [19]	Proposed
Total no. of components	4	13	12	8	9	8	11	13
No. of transistors	1	1	1	1	1	1	1	1
No. of diodes	1	5	3	3	3	2	4	5
System order	2	6	8	4	4	5	5	6
Static Conversion Ratio (M)	D	$D^{3}$	$\frac{n\cdot D}{\left(1+2\cdot D\right)}$	D·(2−D)	$D^{2}$	$\frac{1}{2-D}$	$\frac{D}{2-D}$	$1-{\left(1-D\right)}^{3}$
Transistor dc current stress	$\frac{P_{o}}{M\cdot V_{g}}$	$\frac{P_{o}}{M\cdot V_{g}}$	$\frac{3\cdot \left(n-2\cdot M\right)}{n}\cdot \frac{P_{o}}{M\cdot V_{g}}$	$\frac{P_{o}}{V_{g}}$	$\frac{P_{o}}{M\cdot V_{g}}$	$2\cdot \left(2\cdot M-1\right)\cdot \frac{P_{o}}{M^{2}\cdot V_{g}}$	$\frac{P_{o}}{M\cdot V_{g}}$	$\frac{P_{o}}{V_{g}}$
Transistor voltage stress	$V_{g}$	$\left(1+\sqrt[{3}]{M}+\sqrt[{3}]{M^{2}}\right)\cdot V_{g}$	$\frac{n-M}{n}\cdot V_{g}$	$V_{g}$	$\left(1+\sqrt{M}\right)\cdot V_{g}$	$M\cdot V_{g}$	$\frac{4\cdot M^{2}}{M+1}\cdot V_{g}$	$V_{g}$
Maximum diode dc current stress	$\frac{1-M}{M}\cdot \frac{P_{o}}{M\cdot V_{g}}$	$\frac{P_{o}}{M\cdot V_{g}}$	$\frac{P_{o}}{M\cdot V_{g}}$	$\frac{P_{o}}{M\cdot V_{g}}$	$\frac{P_{o}}{M\cdot V_{g}}$	$\frac{1-M}{M}\cdot \frac{P_{o}}{M\cdot V_{g}}$	$\frac{P_{o}}{M\cdot V_{g}}$	$\sqrt[{3}]{1-M}\cdot \frac{P_{o}}{M\cdot V_{g}}$
Maximum diode voltage stress	$V_{g}$	$\left(1+\sqrt[{3}]{M}\right)\cdot V_{g}$	$\frac{2\cdot M}{n}\cdot V_{g}$	$V_{g}$	$V_{g}$	$M\cdot V_{g}$	$\frac{M+1}{2}\cdot V_{g}$	$V_{g}$

## Data Availability

Data are contained within the article.
